# Beta cell adaptation to pregnancy requires prolactin action on both beta and non-beta cells

**DOI:** 10.1038/s41598-021-89745-9

**Published:** 2021-05-14

**Authors:** Vipul Shrivastava, Megan Lee, Daniel Lee, Marle Pretorius, Bethany Radford, Guneet Makkar, Carol Huang

**Affiliations:** 1 Department of Biochemistry and Molecular Biology, Cumming School of Medicine, University of Calgary, Calgary, T2N 1N4 Canada; 2 Department of Pediatrics, Cumming School of Medicine, University of Calgary, Calgary, T2N 1N4 Canada; 3 Health Science Centre, Room 2219, 3330 Hospital Drive NW, Calgary, AB T2N 4N1 Canada

**Keywords:** Diabetes, Physiology

## Abstract

Pancreatic islets adapt to insulin resistance of pregnancy by up regulating β-cell mass and increasing insulin secretion. Previously, using a transgenic mouse with global, heterozygous deletion of prolactin receptor (Prlr^+/−^), we found Prlr signaling is important for this adaptation. However, since Prlr is expressed in tissues outside of islets as well as within islets and prolactin signaling affects β-cell development, to understand β-cell-specific effect of prolactin signaling in pregnancy, we generated a transgenic mouse with an inducible conditional deletion of Prlr from β-cells. Here, we found that β-cell-specific Prlr reduction in adult mice led to elevated blood glucose, lowed β-cell mass and blunted in vivo glucose-stimulated insulin secretion during pregnancy. When we compared gene expression profile of islets from transgenic mice with global (Prlr^+/−^) versus β-cell-specific Prlr reduction (βPrlR^+/−^), we found 95 differentially expressed gene, most of them down regulated in the Prlr^+/−^ mice in comparison to the βPrlR^+/−^ mice, and many of these genes regulate apoptosis, synaptic vesicle function and neuronal development. Importantly, we found that islets from pregnant Prlr^+/−^ mice are more vulnerable to glucolipotoxicity-induced apoptosis than islets from pregnant βPrlR^+/−^ mice. These observations suggest that down regulation of prolactin action during pregnancy in non-β-cells secondarily and negatively affect β-cell gene expression, and increased β-cell susceptibility to external insults.

## Introduction

Pregnancy is characterized by insulin resistance, to shunt nutrients from the mother to the developing fetus. Maternal pancreatic β-cells adapt to the insulin resistance of pregnancy by up regulating islet mass and insulin secretion, a process that quickly reverses at parturition^1–3^. In animal studies, pregnancy-induced β-cell adaptation consists of (1) a lower threshold for glucose-stimulated insulin secretion, (2) a higher insulin content, and (3) a higher β-cell proliferation rate^4–7^. Both in vitro and in vivo observations support a role for prolactin (PRL) and/or placental lactogens (PLs) in this adaptive process. First, prolactin receptor (Prlr), the receptor for both PRL and PLs, is present on pancreatic β-cells, and Prlr expression increases during pregnancy^3,8,9^. Second, the rise in the levels of PRL and PLs parallels the increases in β-cell mass and glucose-stimulated insulin secretion during pregnancy^3^. Third, in vitro exposure of isolated islets to PRL/PLs increases insulin secretion and β-cell proliferation, and lowers the threshold of glucose-stimulated insulin secretion^10,11^, mimicking the effects of pregnancy on β-cells. To determine whether Prlr signaling is required for β-cell adaptation to pregnancy in vivo, transgenic mice with global or β-cell-specific Prlr knockout have been examined. Using heterozygous global prolactin receptor-null mice (Prlr^+/−^), we established the important in vivo role of Prlr on β-cell adaptation to pregnancy^12^. We found that in comparison to their wild type littermates, pregnant Prlr^+/−^ dams were glucose intolerant and secreted less insulin in response to an intraperitoneal glucose tolerance test (IPGTT). They also had a lower β-cell proliferation rate and lower β-cell mass than the wild type (i.e. Prlr^+/+^) mice^12^. We could not use the homozygous Prlr^−/−^ mice because they have a placental implantation defect, thus are infertile^13^. Two separate groups have since generated β-cell-specific Prlr-knockout mice, one using the rat insulin promoter (RIP) controlled Cre (i.e. RIP-Cre)^14^ and the other using the pancreatic and duodenal homeobox 1 (Pdx1) promoter controlled Cre (i.e. Pdx1-Cre)^15^. Similar to our findings in the Prlr^+/−^ mice, β-cell-specific Prlr-deletion caused gestational diabetes, accompanied by a reduction in β-cell proliferation and a blunted glucose-stimulated insulin secretion in vivo, which is largely due to the lower β-cell mass rather than an intrinsic insulin secretion defect of the islets. However, since both insulin promoter and Pdx1 promoter are expressed in the developing pancreas and embryonic deletion of Prlr causes a reduction in β-cell mass at birth^16^, we generated an inducible conditional Prlr knockout mouse to circumvent this developmental defect. Furthermore, since Prlr is widely expressed^17–20^, including in insulin sensitive tissues such as the skeletal muscles, adipocytes, and hepatocytes, as well as in neurons, epithelial and endothelial cells within islets, the β-cell phenotype observed in Prlr^+/−^ mice might be confounded by the reduction in prolactin action in non-β-cells within and outside of islets. Our objective is to determine the role of prolactin in β-cell adaptation to pregnancy, and to determine whether prolactin action in cells other than β-cells has an effect on β-cell gene expression and survival.

## Results

### Generation of inducible, pancreas-specific prolactin receptor deleted transgenic mice

**Figure 1:**
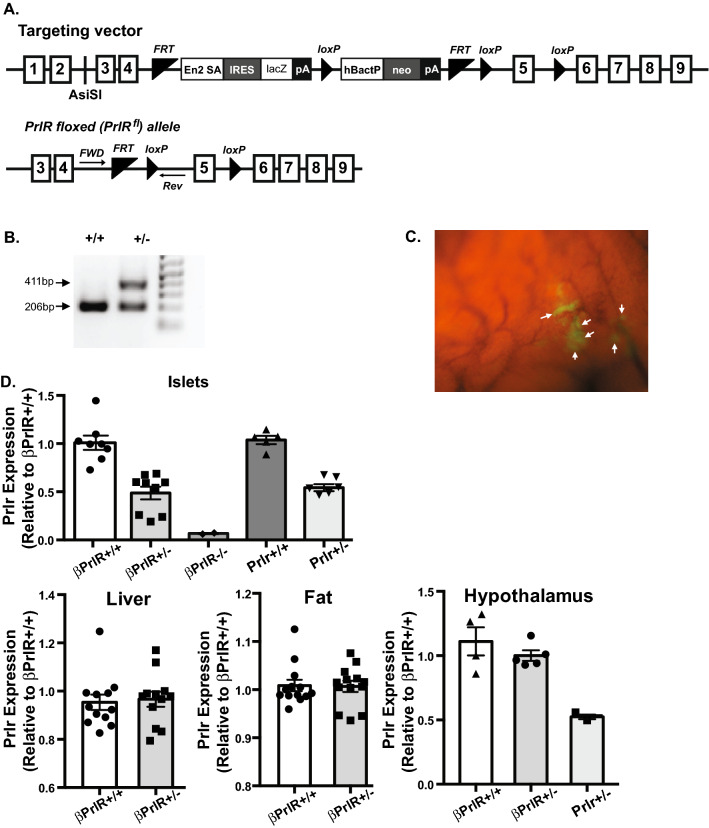
Conditional deletion of prolactin receptor (Prlr) from pancreatic β-cells. (**A**) Schematic diagram of the targeting vector and the floxed prolactin receptor, PrlR, allele. (**B**) Genotyping of litters that contain mice with heterozygous floxed PrlR allele (+/-) or homozygous wild type (non-floxed) PrlR allele (+/+). A subset of the result (mice #284 and 285) is shown here. The entire gel containing the genotyping result is presented in Supplementary Fig. S1. (**C**) Image of pancreas from a βPrlR^−/−^ mouse expressing mT/mG, which allows detection of Cre recombination, visualized as green fluorescence (arrows). The areas of green fluorescence represent islets. (**D**) Relative mRNA expression of prolactin receptor in islets, liver, fat, and hypothalamus. n = 3–12 mice/genotype. “*” = p < 0.05 and “**” = p < 0.01 in comparison to the βPrlR^+/+^ mice. Prlr^+/−^ and Prlr^+/+^ denote transgenic mice with a global, heterozygous deletion of prolactin receptor or their wild type littermates, respectively. βPrlR^−/−^ and βPrlR^+/−^ denote transgenic mice with a β-cell-specific homozygous or heterozygous prolactin receptor deletion, while βPrlR^+/+^ denotes their wild type littermates.

To understand the role of prolactin receptor signaling on pancreatic β-cell mass and function during pregnancy, we generated mice harboring an inducible conditional deletion of Prlr from the pancreatic β-cells (Fig. 1A,B). We interbred the Pdx1CreER mouse, which has a tamoxifen-inducible Cre recombinase under the control of Pdx1, with PrlR^−/−^ mouse, which has a floxed deletion of exon 5 of prolactin receptor, to generate the Pdx1CreER: PrlR^+/−^ mouse, or the βPrlR^+/−^ mouse. To allow visual determination of the tissue-specific prolactin receptor deletion, we intercrossed the βPrlR^+/−^ mouse with the ROSA^mT/mG^ indicator mouse. The ROSA^mT/mG^ mouse constitutively expresses a tdTomato transgene and upon Cre recombination, GFP becomes expressed in the islets (Fig. 1C); we observed no GFP expression in the brain (data not shown). To induce Cre recombinase expression, mice were given tamoxifen at age 8–10 weeks. We found that in comparison to control littermate without the floxed Prlr allele, i.e. the βPrlR^+/+^ mice, the βPrlR^+/−^ mice had a ~ 40% reduction in prolactin receptor expression in isolated islets (Fig. 1D). In comparison to their respective wild type littermates, we found a similar magnitude of reduction in prolactin receptor expression in the islets of βPrlR^+/−^ and Prlr^+/−^ mice, the latter are transgenic mice with a global, heterozygous prolactin receptor deletion, which we have previously reported^12^. The reduction in prolactin receptor is specific to the pancreatic islets, as we observed no significant reduction in prolactin receptor expression in the hypothalamus, fat, and liver (Fig. 1D), tissues that play important roles in glucose homeostasis.

### Gestational glucose intolerance in βPrlR^+/−^ mice

**Figure 2:**
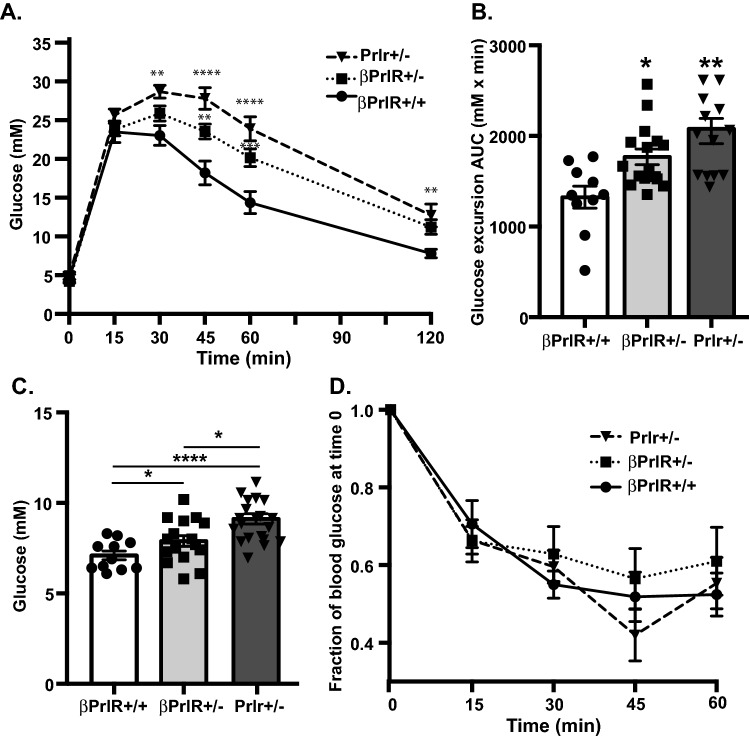
βPrlR^+/−^ and Prlr^+/−^ mice are glucose intolerant during pregnancy. (**A**) Glucose excursion during an intraperitoneal glucose tolerance test. (**B**) Integrated area under the curve (AUC) of glucose excursion during an 120-min IPGTT. (**C**) Random non-fasting blood glucose. (**D**) Glucose excursion during an intraperitoneal insulin tolerance. Blood glucose was expressed as a fraction of the blood glucose at time 0. “*” = p < 0.05, “**” = p < 0.01, and “****” = p < 0.001 in comparison to the wild type βPrlR^+/+^ mice by 2 way ANOVA (**A**, **D**) and 1 way ANOVA (**B**, **C**) with Tukey’s multiple comparisons tests. n = 3–19 mice per genotype.

Next, we determined glucose homeostasis of the βPrlR^+/−^ mice during pregnancy. Intraperitoneal glucose tolerance test (IPGTT) was performed on day 15 of pregnancy. In comparison to their wild type littermates (i.e. βPrlR^+/+^ mice), the βPrlR^+/−^ mice were glucose intolerant with a greater glucose excursion, expressed as integrated area under the curve over the 120 min of the IPGTT (βPrlR^+/−^: AUC = 1770 ± 85.21 mM × min vs. βPrlR^+/+^: AUC = 1325 ± 120.2 mM × min, n = 10–16)(Fig. 2A,B). Interestingly, transgenic mice heterozygous for global prolactin receptor deletion, Prlr^+/−^, had even higher blood glucose at 45 and 60 min of the IPGTT than the βPrlR^+/−^ mice. Non-fasted blood glucose on day 14 of pregnancy is also higher in the βPrlR^+/−^ mice than their wild type littermates (8.09 ± 0.30 mM vs. 7.0 ± 0.24 mM for βPrlR^+/+^ mice) (Fig. 2C), and it is higher still in the Prlr^+/−^ mice (9.17 ± 0.29 mM), although there is no significant difference in fasting blood glucose amongst the 3 genotypes (data not shown). Of note, the presence of Cre recombinase and the administration of tamoxifen per se had no effect on glucose homeostasis, as the βPrlR^+/+^ and the floxed-PrlR mice (which do not have Cre recombinase and never received tamoxifen) had comparable glucose excursion during an IPGTT, with AUC of 1325 ± 120.2 mM × min and 1325.3 ± 261.9 mM × min, respectively. We did not observe a significant difference in glucose response during an insulin tolerance test amongst the βPrlR^+/+^, βPrlR^+/−^, and the Prlr^+/−^ mice on day 14 of pregnancy (Fig. 2D); therefore, the difference in glucose tolerance observed was not a result of difference in insulin sensitivities.

### βPrlR^+/−^ mice had lower β-cell mass and secreted less insulin

**Figure 3:**
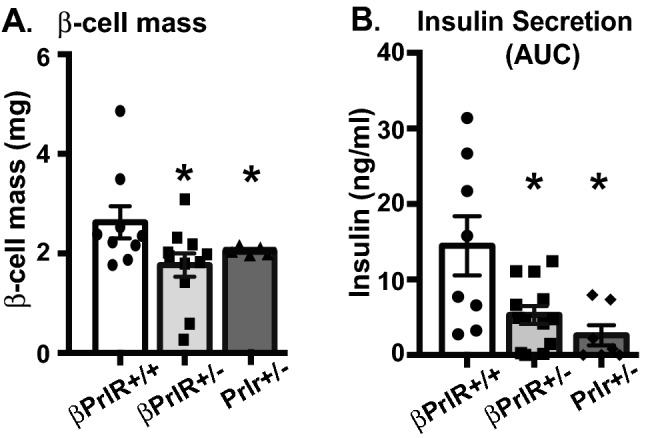
Effect of Prlr deletion on β-cell mass and insulin secretion. (**A**) βPrlR^+/−^ and Prlr^+/−^ mice have a lower β-cell mass on day 15 of pregnancy in comparison to the wild type βPrlR^+/+^ mice. (**B**) Insulin secreted during an IPGTT, expressed as integrated area under the curve (AUC), is lower in βPrlR^+/−^ and Prlr^+/−^ mice in comparison to wild type βPrlR^+/+^ mice. “*” = p < 0.05 in comparison to the βPrlR^+/+^ mice. n = 10–12 mice/genotype.

Similar to our previous observation in the heterozygous global Prlr-deletion mice (Prlr^+/−^)^12^, βPrlR^+/−^ mice had a lower β-cell mass in comparison to their wild type littermates during pregnancy (Fig. 3A). During an IPGTT, both the βPrlR^+/−^ and Prlr^+/−^ mice secreted less insulin in comparison to the wild type mice (Fig. 3B). This blunted in vivo glucose-stimulated insulin secretion is not likely due to an intrinsic insulin secretory defect because we found that islets from βPrlR^+/+^ and βPrlR^+/−^ mice were equally responsive to glucose in vitro, suggesting that the difference in serum insulin levels during an IPGTT likely reflects the smaller β-cell mass found in the mutant mice.

### Prolactin receptor has non-cell-autonomous effects on β-cell gene expression during pregnancy

**Figure 4:**
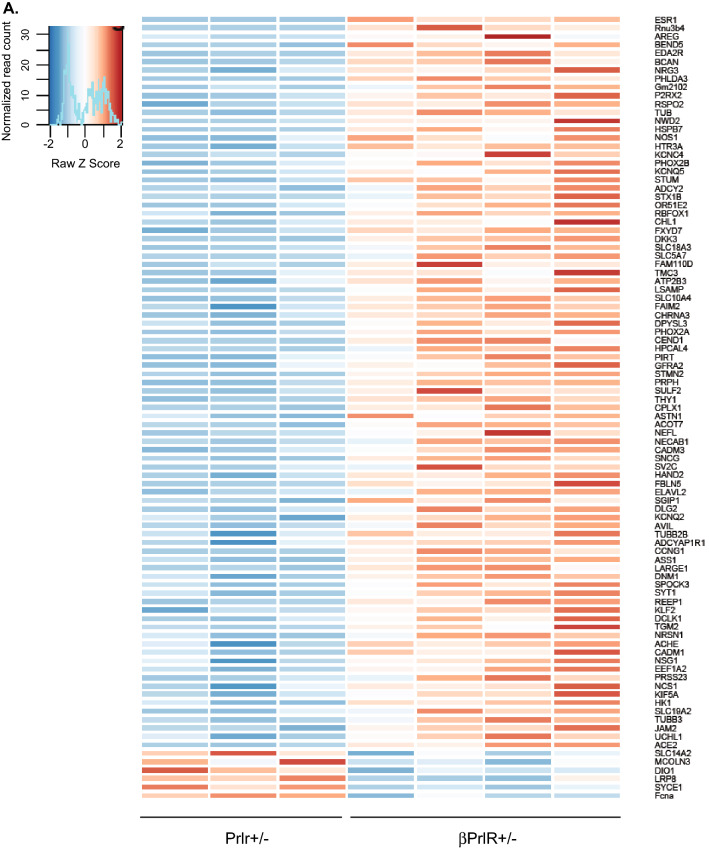
Heat map representing differentially expressed genes in islets from day 15 pregnant Prlr^+/−^ versus βPrlR^+/−^ mice.

**Figure 5:**
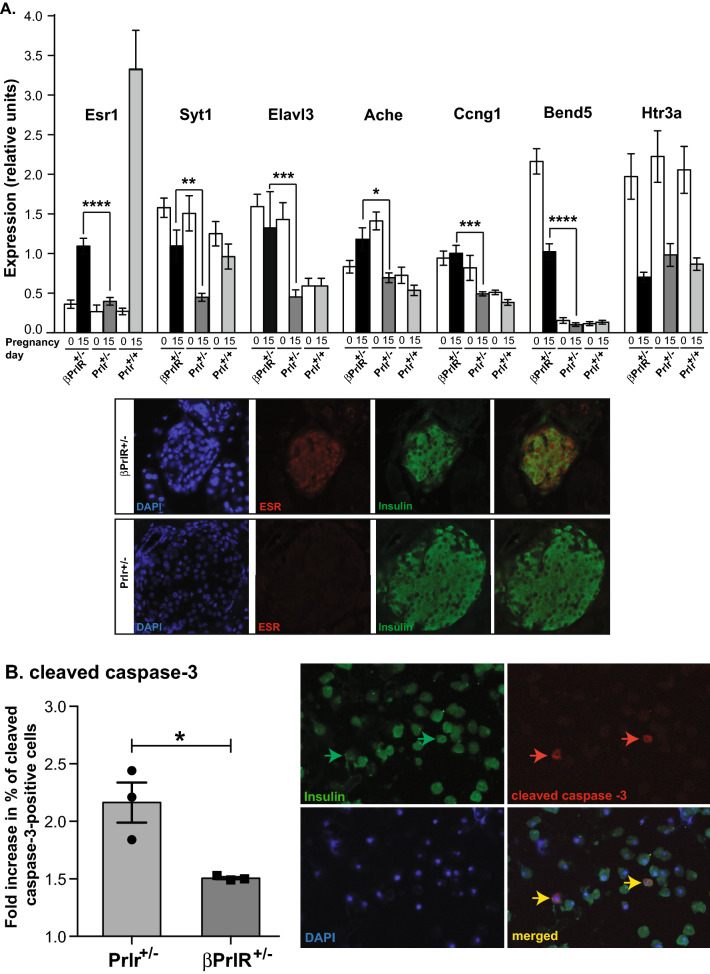
Pancreatic islets from pregnant Prlr^+/−^ mice are more susceptible to apoptosis than those from βPrlR^+/−^ mice. (**A**) Gene expression was determined by RT-qPCR in islets from non-pregnant and pregnant (day 15) βPrlR^+/−^, Prlr^+/−^ and Prlr ^+/+^ mice. N = 6–13 mice per genotype at day 0 or day 15 of pregnancy. “****” = p < 0.001, “***” = p < 0.005, “**” = p < 0.01, and “*” = p < 0.05. Representative images of β-cell and ERα co-staining in an islet in βPrlR^+/−^ or Prlr^+/−^ mouse are presented. (**B**) Islets from day 15 pregnant Prlr^+/−^ and βPrlR^+/−^ mice were treated with 0.5 mM of palmitate or BSA control for 72 h, then immunostained for insulin and cleaved caspase-3. Results are expressed as the fold-increase in the percentage of cleaved caspase-3 positive β-cells in the palmitate-treated islets relative to the BSA-treated islets within each genotype. N = 3 mice from each genotype. At least 5000 cells were counted for each mouse. “*” = p < 0.05 in comparison to Prlr^+/−^ mice. A representative image of cells stained for insulin (green), cleaved caspase-3 (red), and nuclear stained with Hoechst is presented. The two yellow arrows in the right lower panel points to two cells that are positive for both insulin (green, upper left panel) and cleaved caspase-3 (red, upper right panel).

The above results demonstrated that transgenic mice with global (Prlr^+/−^) or β-cell-specific prolactin receptor deletion (βPrlR^+/−^) are phenotypically similar in the metabolic parameters measured: in comparison to their wild type littermates, both mutant mice are glucose intolerant on day 15 of pregnancy, with a lower β-cell mass, which leads to a lower amount of insulin being secreted in response to a glucose load, supporting the functional importance of prolactin receptor in islet adaptation to pregnancy. To understand the effect of Prlr action in non-β-cells vis-à-vis β-cells in regulating β-cell adaptation to pregnancy, we compared gene expression profile of islets from Prlr^+/−^ to that of βPrlR^+/−^ mice. It is important to note that in Prlr^+/−^ mice, the reduction in Prlr expression would be widespread, including β-cells as well as non-β-cells within the islets such as endothelial cells that lines the vasculature and neurons that innervates the islets. Furthermore, Prlr expression is also reduced in tissues outside of islets, including but not limited to tissues such as the hypothalamus, muscle, fat, and liver^20^. Using Sleuth^21^ and a false discovery rate (FDR) threshold of 0.05, we identified 95 differentially expressed genes in islets (Table 1 and Fig. 4); most of them are down regulated in the Prlr^+/−^ mice in comparison to the βPrlR^+/−^ mice, with estrogen receptor 1 (ESR1) being the most significantly affected gene (Fig. 5A). Since estrogen receptor is known to be expressed in β-cells in islets and activation of estrogen receptor has been shown to protect β-cells from apoptosis^22^, we compared the susceptibility of islets isolated from the pregnant Prlr^+/−^ and βPrlR^+/−^ mice to fatty-acid-mediated induction of apoptosis in vitro^22–24^. We treated isolated islets with 0.5 mM of palmitate or BSA control for 72 h. Using cleaved caspase-3 positivity as a marker of activation of the apoptosis pathway, we found that palmitate induced a greater increase in the percentage of insulin-positive cells that are also positive for cleaved caspase-3 in islets from Prlr^+/−^ mice in comparison to islets from βPrlR^+/−^ mice (Fig. 5B). This is consistent with the pro-survival role of the estrogen receptor, which is expressed at a lower level in islets of pregnant Prlr^+/−^ mice than in βPrlR^+/−^ mice. This suggests that the down regulation of Prlr action in non-β-cells within and outside islets, as would be expected in the Prlr^+/−^ mice, increased β-cell susceptibility to apoptosis.

**Table 1:** Gene expression levels in islets from day 15 pregnant Prlr^+/−^ mice relative to βPrlR^+/−^ mice.

Entrez gene name	Gene symbol	Expression log ratio	False discovery rate (q-value)
Estrogen receptor 1	ESR1	− 5.686	8.92E − 294
U3B small nuclear RNA 4	Rnu3b4	− 4.552	1.65E − 09
Amphiregulin	AREG	− 1.624	0.0494
BEN domain containing 5	BEND5	− 1.57	1.77E − 09
Ectodysplasin A2 receptor	EDA2R	− 1.324	0.000000236
Brevican	BCAN	− 1.263	0.00367
Neuregulin 3	NRG3	− 1.168	0.0322
Pleckstrin homology like domain family A member 3	PHLDA3	− 1.158	1.84E − 16
Inka box actin regulator 2	FAM212B	− 0.977	2.92E − 13
Purinergic receptor P2X 2	P2RX2	− 0.891	0.021
R-spondin 2	RSPO2	− 0.866	0.00753
TUB bipartite transcription factor	TUB	− 0.862	0.00976
NACHT and WD repeat domain containing 2	NWD2	− 0.86	0.00533
Heat shock protein family B (small) member 7	HSPB7	− 0.857	0.00432
Nitric oxide synthase 1	NOS1	− 0.855	0.0209
5-Hydroxytryptamine receptor 3A	HTR3A	− 0.847	0.0000846
Potassium voltage-gated channel subfamily C member 4	KCNC4	− 0.83	0.00349
Paired like homeobox 2B	PHOX2B	− 0.823	0.000808
Potassium voltage-gated channel subfamily Q member 5	KCNQ5	− 0.813	0.011
Stum, mechanosensory transduction mediator homolog	STUM	− 0.798	0.00000442
Adenylate cyclase 2	ADCY2	− 0.797	0.00403
Syntaxin 1B	STX1B	− 0.788	0.00161
Olfactory receptor family 51 subfamily E member 2	OR51E2	− 0.786	0.0000263
RNA binding fox-1 homolog 1	RBFOX1	− 0.785	0.0202
Cell adhesion molecule L1 like	CHL1	− 0.78	0.00432
FXYD domain containing ion transport regulator 7	FXYD7	− 0.773	0.04
dickkopf WNT signaling pathway inhibitor 3	DKK3	− 0.766	0.000001
Solute carrier family 18 member A3	SLC18A3	− 0.763	0.0000104
Solute carrier family 5 member 7	SLC5A7	− 0.76	0.0000104
Family with sequence similarity 110 member D	FAM110D	− 0.757	0.0195
Transmembrane channel like 3	TMC3	− 0.753	0.0327
ATPase plasma membrane Ca^2+^ transporting 3	ATP2B3	− 0.733	0.0174
Limbic system associated membrane protein	LSAMP	− 0.731	0.0000039
Solute carrier family 10 member 4	SLC10A4	− 0.722	0.000929
Fas apoptotic inhibitory molecule 2	FAIM2	− 0.719	0.00753
Cholinergic receptor nicotinic alpha 3 subunit	CHRNA3	− 0.718	0.000188
Dihydropyrimidinase like 3	DPYSL3	− 0.705	0.0000121
Paired like homeobox 2A	PHOX2A	− 0.705	0.031
Cell cycle exit and neuronal differentiation 1	CEND1	− 0.689	0.0178
Hippocalcin like 4	HPCAL4	− 0.675	0.00744
Phosphoinositide interacting regulator of transient receptor potential channels	PIRT	− 0.663	0.00234
GDNF family receptor alpha 2	GFRA2	− 0.655	0.0214
Stathmin 2	STMN2	− 0.653	1.77E − 09
Peripherin	PRPH	− 0.649	0.000000785
Sulfatase 2	SULF2	− 0.647	0.000188
d-Glutamate cyclase	C14orf159	− 0.643	1.83E − 08
Thy-1 cell surface antigen	THY1	− 0.64	0.00000425
Complexin 1	CPLX1	− 0.639	0.00108
Astrotactin 1	ASTN1	− 0.636	0.0327
Acyl-CoA thioesterase 7	ACOT7	− 0.62	0.000246
Neurofilament light	NEFL	− 0.62	0.0118
N-terminal EF-hand calcium binding protein 1	NECAB1	− 0.604	0.021
Cell adhesion molecule 3	CADM3	− 0.596	0.000949
Synuclein gamma	SNCG	− 0.581	0.00000425
Synaptic vesicle glycoprotein 2C	SV2C	− 0.572	0.0202
Heart and neural crest derivatives expressed 2	HAND2	− 0.571	0.0157
Fibulin 5	FBLN5	− 0.565	0.00318
ELAV like RNA binding protein 2	ELAVL2	− 0.556	0.0161
SH3GL interacting endocytic adaptor 1	SGIP1	− 0.543	0.0426
Discs large MAGUK scaffold protein 2	DLG2	− 0.541	0.00172
Potassium voltage-gated channel subfamily Q member 2	KCNQ2	− 0.539	0.00478
Advillin	AVIL	− 0.523	0.0152
Tubulin beta 2B class IIb	TUBB2B	− 0.511	0.0211
ADCYAP receptor type I	ADCYAP1R1	− 0.509	0.00227
Cyclin G1	CCNG1	− 0.49	0.00000439
Argininosuccinate synthase 1	ASS1	− 0.489	0.000000027
LARGE xylosyl- and glucuronyltransferase 1	LARGE1	− 0.474	0.027
Dynamin 1	DNM1	− 0.464	0.0000284
SPARC (osteonectin), cwcv and kazal like domains proteoglycan 3	SPOCK3	− 0.461	0.00454
Synaptotagmin 1	SYT1	− 0.458	0.0000483
Receptor accessory protein 1	REEP1	− 0.441	0.0188
Kruppel like factor 2	KLF2	− 0.435	0.00309
Doublecortin like kinase 1	DCLK1	− 0.414	0.00367
Transglutaminase 2	TGM2	− 0.41	0.00626
Neurensin 1	NRSN1	− 0.403	0.00323
Acetylcholinesterase (Cartwright blood group)	ACHE	− 0.402	0.0152
Cell adhesion molecule 1	CADM1	− 0.373	0.0333
Neuronal vesicle trafficking associated 1	NSG1	− 0.37	0.0497
Eukaryotic translation elongation factor 1 alpha 2	EEF1A2	− 0.36	0.000472
Serine protease 23	PRSS23	− 0.358	0.0127
Neuronal calcium sensor 1	NCS1	− 0.357	0.0353
Kinesin family member 5A	KIF5A	− 0.354	0.00571
Hexokinase 1	HK1	− 0.341	0.0332
Solute carrier family 19 member 2	SLC19A2	− 0.34	0.0365
Tubulin beta 3 class III	TUBB3	− 0.32	0.00274
Junctional adhesion molecule 2	JAM2	− 0.313	0.00626
Ubiquitin C-terminal hydrolase L1	UCHL1	− 0.287	0.0278
Angiotensin I converting enzyme 2	ACE2	− 0.278	0.0107
Solute carrier family 14 member 2	SLC14A2	0.439	0.0227
Mucolipin 3	MCOLN3	0.517	0.00626
Iodothyronine deiodinase 1	DIO1	0.63	0.0000092
LDL receptor related protein 8	LRP8	0.698	0.000095
Synaptonemal complex central element protein 1	SYCE1	1.026	0.000138
Ficolin A	Fcna	1.112	0.00403

Next, using Ingenuity Pathway Analysis, we identified several canonical pathways that are differentially expressed, including citrulline cycle and metabolism, dopamine receptor signaling, synaptogenesis signaling pathway and the serotonin receptor-signaling pathway, the latter has previously been found to regulate β-cell proliferation during pregnancy^25^ (Table 2). KEGG pathway analysis of the 95 genes identified enrichment of genes involved in cholinergic synapse (p = 3.39 × 10^–4^) and synaptic vesicle cycle (p = 4.96 × 10^–3^) (Table 2). GO term analysis found enrichment in genes that are involved in synaptic transmission (p = 6.45 × 10^–4^), nervous system development (p = 6.03 × 10^–4^), anterograde trans-synaptic signaling (p = 3.62 × 10^–3^), noradrenergic neuron differentiation (p = 4.57 × 10^–3^), and cell differentiation in hindbrain (p = 2.26 × 10^–2^) (Table 2). Using RT-qPCR, we confirmed that acetylcholinesterase (Ache) and synaptotagmin 1 (Syt1), both identified in the KEGG pathway and GO term analysis (Table 2), expressed at a lower level in islets of pregnant PrlR^+/−^ mice in comparison to the βPrlR^+/−^ mice. In addition, BEN domain containing 5 (Bend5), a transcription repressor, cyclin G1 (Ccng1), which regulates cell cycle and growth, and ELAV like RNA binding protein 3 (Elavl3), a neural-specific RNA-binding protein, also expressed at a lower level in islets from pregnant Prlr^+/−^ mice in comparison to the βPrlR^+/−^ mice (Fig. 5A). These differences in gene expression between the pregnant Prlr^+/−^ and βPrlR^+/−^ were not due to difference in developmental expression prior to pregnancy because except for Bend5, we did not detect a significant difference in expression of these genes between non-pregnant Prlr^+/−^ and βPrlR^+/−^ mice. One common theme from these pathway analyses is the down regulation of genes that regulate neuronal structure and function as a result of global down regulation of prolactin receptor. This is highly interesting because previously, prolactin receptor action was shown to be important in neurogenesis in the forebrain subventricular zone of female mice during pregnancy, an area of the brain that regulates maternal behavior^26^.

**Table 2:** Pathway analysis of the 95 differentially expressed genes between islets of pregnancy Prlr^+/−^ and βPrlR^+/−^ mice.

A			
Canonical pathways	Overlap	Genes	p-value
Citrulline-nitric oxide cycle	2/5	*ASS1, NOS1*	9.83^−5^
Serotonin receptor signaling	3/43	*ADCY2, HTR3A, SLC18A3*	3.43^−4^
Synaptogenesis signaling pathway	6/327	*ADCY2, CPLX1, LRP8, SNCG, STX1B, SYT1*	5.94^−4^
Superpathways of citrulline metabolism	2/15	*ASS1, NOS1*	1.01^−3^
Dopamine Receptor Signaling	3/77	*ADCY2, NCS1, SLC18A3*	1.88^−3^

B			
KEGG pathways	Overlap	Enriched genes	p-value
Cholinergic synapse	7/113	*ACHE, SLC5A7, CHRNA3, KCNQ2, KCNQ5, ADCY2,SLC18A3*	3.39^−4^
Synaptic vesicle cycle	5/77	STX1B, SYT1, CPLX1, DNM1, SLC18A3	4.96^−3^

C			
GO (biological process)	Overlap	Enriched genes	p-value
Chemical synaptic transmission	11/289	*SLC5A7, CHRNA3, DLG2, SYT1, KCNC4, KIF5A, KCNQ2, HTR3A,NSG1, CPLX1, SLC18A3*	6.45^−4^
Nervous system development	13/455	*ACHE, BCAN, RBFOX1, CHRNA3, NRSN1, AVIL, LSAMP, HPCAL4, GFRA2, PHOX2B, DCLK1, DLG2, KCNQ2*	6.03^−4^
Anterograde trans-synaptic signaling	9/240	*CHRNA3, DLG2, SYT1, KCNC4, KIF5A, KCNQ2, HTR3A, CPLX1, SLC18A3*	3.62^−3^
Noradrenergic neuron differentiation	3/7	*HAND2, PHOX2B, PHOX2A*	4.57^−3^
Cell differentiation in hindbrain	3/12	*CEND1, FAIM2, PHOX2B*	2.26^−2^

(A) Canonical pathways that are differentially expressed in Prlr^+/−^ versus βPrlR^+/−^ mice, as identified by the Ingenuity Pathway Analysis. (B) KEGG pathway analysis of the 95 genes (from Table 1) identified 2 significantly enriched pathways (https://maayanlab.cloud/Enrichr/enrich#). (C) GO term analysis of the 95 genes (from Table 1) identified 5 significantly enriched pathways.

## Discussion

Pregnancy is an insulin resistant state and maternal pancreatic islets adapt to the increase in insulin demand by up regulating β-cell mass and increasing insulin secretion. Previously, we showed that a transgenic mouse with a global, heterozygous deletion of prolactin receptor (Prlr^+/−^) was glucose intolerant during pregnancy. This was accompanied by a reduction in β-cell mass and a blunted glucose-stimulated insulin secretion in vivo, commensurate with the lower β-cell mass^12^. A caveat of using a global knockout mouse is that the prolactin receptor is widely expressed^18,27^, raising the possibility that the phenotype observed in Prlr^+/−^ mice may be secondary to prolactin action in cells other than β-cells. To address this possibility, we generated a transgenic mouse with an inducible, conditional prolactin receptor gene knockout. We chose to make the gene deletion inducible because β-cell-specific promoters such as the insulin promoter or the Pdx1 promoter are active during embryonic development of the pancreas^28^. In fact, Auffret et al. found that transgenic mice with a global homozygous deletion of prolactin receptor (i.e. Prlr^−/−^) have abnormal β-cell mass expansion both embryonically and during perinatal life^16^. They found a 30% reduction in β-cell mass in the Prlr^−/−^ newborns. A transgenic mouse with an inducible promoter such that the prolactin receptor gene is only deleted during adulthood would circumvent this developmental defect and allow us to study prolactin receptor’s effect specifically during pregnancy. Here, we report that in βPrlR^+/−^ mice, the deletion of prolactin receptor in adulthood, before pregnancy, led to maladaptation to insulin resistance of pregnancy, with a lower β-cell mass and insulin secretion, as well as a higher blood glucose level in the βPrlR^+/−^ mice in comparison to their wild type littermates. As expected, β-cell-specific deletion of prolactin receptor did not have a significant effect on overall insulin sensitivity, while the Prlr^+/−^ mice had a tendency for slightly lower insulin sensitivity at 45 min of the ITT (Fig. 2D). This tendency to increased insulin sensitivity however, is not likely to be a significant driver of the lower β-cell mass and insulin secretion in the Prlr deficient mice because if the lower insulin secretion observed in the Prlr^+/−^ mice reflected their improved insulin sensitivity thereby a reduced need for insulin^29^, we would anticipate Prlr^+/−^ mice to have lower blood glucose than βPrlR^+/−^ mice. Our results showed that both Prlr^+/−^ and βPrlR^+/−^ have higher blood glucose than the wild type mice and in fact, Prlr^+/−^ mice had slightly higher, not lower blood glucose than βPrlR^+/−^ mice. Therefore, the lower insulin sensitivity in the Prlr^+/−^ mice at 45 min of ITT is not likely a significant determinant of β-cell mass and insulin secretion.

Other groups have since generated conditional knockouts of the prolactin receptor gene. Banerjee et al. used a RIP-Cre promoter to generate a conditional Prlr knockout^14^. They found that β-cell-specific deletion of Prlr led to gestational diabetes due to reduced β-cell proliferation and a failure to expand β-cell mass during pregnancy. They identified MafB as one of the Prlr-signaling targets, and MafB deletion in maternal β-cells caused gestational diabetes as well. Subsequently, they performed a transcriptome analysis and found that forkhead box protein M1, polycomb repressor complex 2 subunits, Suz12 and enhancer of zeste homolog 2 are additional Prlr signaling targets^30^. Interestingly, Prlr was not required for β-cell adaptation to high fat feeding. In fact, pregnancy and high fat feeding activate very different genes in the islets, suggesting that the two metabolic stressors engage different mechanisms to adapt. Nteeba et al. also generated a β-cell-specific Prlr knockout mouse, using a Pdx1-Cre promoter^15^. In agreement with Banerjee et al., they found glucose intolerance during pregnancy. Interestingly, the β-cell-specific Prlr knockout mice had lower β-cell mass in the non-pregnant state and the pregnancy-stimulated β-cell mass expansion was essentially non-existent in their model. Moreover, they found that the loss of maternal pancreatic Prlr signaling was associated with fetal overgrowth and dysregulation of prolactin-associated genes in the placenta. The functional consequence of the placental findings was not explored in that study, but it is of high interest because we previously reported that Prlr deletion had deleterious, transgenerational effect such that the wild type offspring from Prlr^+/−^ dams had maladaptive β-cell responses during pregnancy^31^, which could be secondary to an abnormal placenta.

Prolactin receptor expression is widespread. Amongst endocrine cells of the pancreatic islet, Prlr expression is limited to β-cells but it is also expressed in endothelial cells that lines the blood vessels and neurons, both are non-β-cells that are present in islets. As well, Prlr is expressed in many non-β-cells outside of islets, such as in the brain, muscles, fat, and liver. To determine whether reduction of Prlr in non-β-cells, including both non-β-cells within islets and those outside of the islets, has an effect on islet gene expression during pregnancy, we performed RNAseq analysis, comparing gene expression of islets of Prlr^+/−^ versus βPrlR^+/−^ mice on day 15 of pregnancy. Interestingly, 95 genes are differentially expressed, and 89 out of 95 are down regulated in the islets of Prlr^+/−^ mice in comparison to βPrlR^+/−^ mice, with estrogen receptor 1 (ESR1) being the most significantly down regulated gene. This is interesting because Le May et al. showed that ERα is expressed in β-cells and activation of estrogen receptor protects β-cells from apoptosis^22–24^. Our previous observation that islets from pregnant mice are more resistant to free fatty acid induced apoptosis than islets from non-pregnant mice^32^ may be in part attributable to the > 20-fold induction of ESR1 expression in islets during pregnancy. Indeed, when we exposed isolated islets from pregnant Prlr^+/−^ and βPrlR^+/−^ mice to free fatty acid, we observed a greater increase in percentage of activated caspase-3 positive β-cells relative to untreated cells in the Prlr^+/−^ islets in comparison to the βPrlR^+/−^ islets. A potential link between Prlr signaling and ESR1 expression is TGFβ, as our previous work on Prlr^+/−^ mice found that TGFβ is up regulated in Prlr^+/−^ mice in comparison to Prlr^+/+^ mice, and TGFβ has been shown to regulate both Prlr and ESR1. Other potential factors that may act in an endocrine or a paracrine fashion to regulate β-cell gene expression include growth hormone^32–34^ and hepatic growth factor^35^, as both are secreted from non-β-cells outside of the islets and expressed in non-β-cells within the islets, such as endothelial cells^32,36,37^, and both hormones have pro-proliferative and pro-survival effects on β-cells. Interestingly, growth hormone has been shown to regulate Syt1 expression^38^, another gene that is expressed at a lower level in the Prlr^+/−^ mice in comparison to the βPrlR^+/−^ mice (Fig. 5). It is important to note that since the RNAseq analysis was performed using whole islets and not purified β-cells, the difference in gene expression may reflect differential gene expression in various cell types in the islets and not only the difference in β-cells. While this is possible, we believe the difference in gene expression most likely reflects expression in β-cells because on day 15 of pregnancy, β-cells constitute 85–90% of endocrine cells in an islet and endothelial cells constitute ~ 10–15% of islet mass while neurons contribute minimally to islet mass (preliminary data not shown). Therefore, β-cells are the most abundant cell type in an islet during pregnancy and the gene expression changes are most likely occurring in β-cells. The β-cell gene expression difference between islets of βPrlR^+/−^ and Prlr^+/−^ mice however, are likely secondary to the down regulation of Prlr in non-β-cells because βPrlR^+/−^ and Prlr^+/−^ mice have comparable Prlr expression in islets (Fig. 1D) but Prlr^+/−^ (and not βPrlr^+/−^) mice also have down regulation of Prlr in non-β-cells, both within and outside of islets. This led us to speculate that there is Prlr-dependent endocrine (from non-β-cells outside of islets) and/or paracrine factors (from non-β-cells within islets) that affect gene expression in β-cells in βPrlr^+/−^ and Prlr^+/−^ mice.

An interesting finding from our RNAseq analysis was the identification of serotonin receptor signaling as one of the top canonical pathways that are differentially regulated between Prlr^+/−^ and βPrlR^+/−^ mice ^25,39^. Kim et al. reported that serotonin acts downstream of Prlr to stimulate β-cell proliferation, and this was through the Gαq-linked serotonin receptor 5-hydroxytryptamine receptor-2b (Htr2b)^25^. In a subsequent study, they found that another serotonin receptor, Htr3a, is important for regulation of insulin secretion^40^, although we did not detect a difference in Htr3a expression between the Prlr^+/−^ and βPrlR^+/−^ mice in this study.

Hence, observations from our β-cell-specific Prlr knockout mice as well as those from Banerjee et al. and Nteeba et al.^14,15^, and the global Prlr knockout mice^12^, indicate that Prlr has cell-autonomous effects on β-cell adaptation to pregnancy, which is accountable for most of the observed Prlr effect on β-cell mass expansion and insulin secretion during pregnancy. The current study reports the novel finding that the absence of Prlr action in non-β-cells increased β-cell’s susceptibility to apoptosis in response to an external insult such as glucolipotoxicity, and that Prlr action in non-β-cells also affect β-cell gene expression during pregnancy, suggesting the presence of Prlr-dependent ‘factor(s)’ from non-β-cells within the islets and/or tissues outside of islets that regulate β-cell adaptation to pregnancy.

## Methods

### Ethical approval

All experimental procedures were approved by the Animal Use Review Committee at the University of Calgary in accordance with standards of the Canadian Council on Animal Care. The study was carried out in compliance with the ARRIVE guidelines.

### Mice

Heterozygous prolactin receptor null mice (Prlr^+/−^) on a C57BL/6 background were purchased from Jackson Laboratory and a working stock was generated by crossing Prlr^+/−^ mice with wild type Prlr^+/+^ mice. The pups were genotyped as previously described^41^. Prlr^+/+^ and Prlr^+/−^ mice did not receive tamoxifen.

### Generation of the conditional prolactin-receptor null mice

A promoter-driven targeting cassette for the generation of a knockout-first allele with potential for conditional knockout was obtained from EUCOMM (The European Conditional Mouse Mutagenesis Program)^42^. The vector was linearized at AsiSI-site and electroporated into mouse ES cells. Neomycin resistant cells were isolated and selected by Southern analysis. ES cells that contain the correctly recombined genome and correct karyotype were then injected into 8-cell CD1E wild type mouse embryos to generate chimeras. The male chimeric mice were then crossed to albino C57BL/6 mice (Charles River, B6N-*Tyr*^C−Brd/BrdCrCrl^) to obtain germline transmission of the mutation. The FRT-flanked neo cassette was removed by crossing with FLPeR mice (The Jackson Laboratories; B6.129S4-*Gt(ROSA)26Sor*^*1(FLP1)Dym*^/RainJ). This generated the mice heterozygous for floxed exon 5 (*Prlr*^*1c*(EUCOMM)Hmgu^, herein denoted as *PrlR*^+/−^) that were phenotypically wild-type. It was back-crossed with C57BL/6J mice (The Jackson Laboratory) for more than 10 generations. The PrlR^+/−^ mice were crossed with Pdx1CreER mouse (from Mutant Mouse Resource & Research Centers supported by NIH, Stock No.: 000350-UCD)^43^, which has the *Cre-ER* gene under the control of the *Pdx1* promoter, to generate the Pdx1CreER:PrlR^+/−^ mice. Pdx1 is expressed in the pancreatic bud during embryonic development and it is required for the formation of pancreas; in adult pancreas, its expression is restricted to the β cells^44^. The male Pdx1CreER:PrlR^+/−^ mice were crossed with female PrlR^+/−^ mice to generate the homozygous conditional knockout Pdx1CreER:PrlR^−/−^ (herein denoted as βPrlR^−/−^), and the heterozygous conditional knockout βPrlR^+/−^, and the control littermates, i.e. Pdx1CreER, PrlR^+/−^ and PrlR^−/−^ mice. The βPrlR^−/−^ mice were then crossed with the mT/mG reporter mice (The Jackson Laboratory, Stock No.: 007576), which carries *ROSA*^*mT/mG*^, a cell membrane-targeted, two-color fluorescent Cre-reporter allele. Before Cre recombination, the tdTomato (mT) fluorescence expression is widespread in all cells. Upon Cre recombinase activation, the cells express membrane-localized EGFP (mG) fluorescence, replacing the red fluorescence ^45^. To induce Cre recombinase activity, tamoxifen (dissolved in corn oil) was given by oral gavage at age 8 weeks for 5 days at a dose of 4 mg/day (for a 20 g mouse). Mice were used 4 weeks after tamoxifen administration.

Mice were maintained on a 12-h light, 12-h dark cycle with liberal access to food and water. Mice were studied at 4–5 months of age. For pregnancy, female mice were set up with wild type male mice and the morning when a vaginal plug was found was designated as day 1 of pregnancy. Mice were used on day 15 of pregnancy. We chose to study day 15 of pregnancy because β-cell proliferation and β-cell mass peaks on days 14–15 of pregnancy^3,9^.

### Intraperitoneal glucose tolerance test (IPGTT) and insulin tolerance test (ITT)

IPGTT (20% d-glucose solution, 2 g/kg body weight) and ITT (NovoRapid insulin, 0.75 unit/kg body weight) were performed as previously described^12^. Additional blood samples (~ 30 µl) were taken at times 0, 5, and 30 min of IPGTT for insulin concentration measurements by ELISA (ALPCO, catalog #80-INSMSU-E01). Non-fasted blood glucose was determined using a glucometer (FastTake) by sampling from tail vein at 8 a.m., and at the same time, an additional 30 μl of serum was taken from the saphenous vein and stored at − 80 °C for later measurement of insulin by ELISA.

### Materials

Collagenase P (Ref#11 213 865 001) was purchased from Roche. Trypsin 1 mg tablets (T7168) and all chemicals are from Sigma. Sources of antibodies are specified below.

### Immunostaining

For quantification of β-cell mass, pancreas was isolated from pregnant and non-pregnant mice, weighed, embedded in paraffin blocks, longitudinally serial sectioned to 7 μm, then stained for insulin to identify β cells as previously described^12^. Briefly, after 1 h of blocking with 1% goat serum/phosphate buffered saline (PBS) at room temperature, tissues were incubated with primary antibody over night at 4 °C (guinea pig anti-insulin at 1:750, DAKO; diluted in 1% goat serum/PBS). This was followed by 1-h incubation with fluorophore-conjugated secondary antibodies (Cy3-anti-guinea pig at 1:300, Jackson ImmunoResearch, diluted in 1% goat serum/PBS). Bis-benzimide H 33342 trihydrochloride (0.1 μg/ml, Sigma) was added to the secondary antibody for nuclear staining. Stained sections were mounted using DakoCytomation fluorescent Mounting Medium (Agilent/Dako, Santa Clara, USA) and stored at 4 °C. To localize estrogen receptor-α (ERα) in pancreatic tissues, antigen retrieval was achieved by microwaving the paraffin embedded tissue sections in sodium citrate (pH 6), followed by 1 h of blocking with 1% goat serum/PBS at room temperature, then incubation with primary antibodies overnight at 4 °C to co-stain for insulin (to identify β-cells) and ERα (rabbit anti-ERα at 1:100, Santa Cruz). Next day, after 3 brief washes in PBS, tissue sections were incubated with secondary antibodies (Cy3-anti-rabbit at 1:300 and Alexa488-anti-guinea pig at 1:300, both diluted in 1% goat serum/PBS, plus Hoechst 33342 for nuclear staining) at room temperature for 1 h. For identification of cleaved caspase-3 positive cells, isolated mouse islets were incubated in 0.5 mM of palmitate in the presence of 33 mM glucose (see below) for 72 h. At the end of the incubation, islets were briefly washed in PBS, dissociated into single cells using 2 mM EDTA, and place on poly-l-lysine coated glass slides. The cells were fixed with 4% PFA in PBS for 10 min at room temperature and permeabilized with 0.2% Triton X for 15 min, then incubated with rabbit cleaved caspase-3 antibody (Promega, Madison, USA) at 1:1000 dilution in 1% goat serum/PBS at 4 °C overnight, followed by incubation with goat-anti-rabbit-Cy3 (Jackson ImmunoResearch, West Grove, USA) or goat-anti-rabbit-Alexa-488 antibodies (Molecular Probes, Waltham, USA) at 1:300 dilution in 1% goat serum/PBS at room temperature for 1 h. After 3 washes with PBS, cells were mounted with Dako fluorescent Mounting Medium.

### Islet morphometry

Consecutive images of non-overlapping, adjacent areas of the entire pancreas section were acquired using a Zeiss fluorescence microscope, and captured with a CoolSnap digital camera^12^. Images were analyzed by ImageJ software (https://imagej.nih.gov/ij/) to measure the insulin-positive area as well as the area of the entire pancreas section (identified by nuclear staining). β-cell mass was calculated by multiplying the pancreas weight by the β-cell fraction (i.e. the ratio of insulin-positive cell area to total pancreatic tissue area on the entire section). Results represent the average of 6–8 tissue sections per animal from 5 to 6 animals from each genotype.

### Islet isolation

Pancreatic islets were isolated from non-fasted adult female mice. The pancreas was first distended using collagenase P (0.66 mg/ml in Hank’s Balanced Salt Solution, 2.5 ml/pancreas), surgically removed and then incubated at 37 °C for 15 min under constant agitation. Islets were hand-picked and cultured overnight in RPMI1640 supplemented with 10% fetal bovine serum (FBS) and penicillin/streptomycin^31^. For some experiments, islets were treated with 0.5 mM palmitate (PA) in the presence of 33 mM d-glucose (total concentration) in RPMI1640 supplemented with fatty-acid free bovine albumin serum (without FBS) for 72 h. Control islets were incubated in culture media containing vehicle (1% fatty-acid free bovine albumin serum and 0.25% ethanol) and the non-metabolizable l-glucose for a final concentration of 33 mM glucose, as a control for osmolality.

### Islet RNA isolation and quantitative real-time RT-PCR

Total islet RNA (100–200 islets/mouse) was extracted using the RNeasy Mini Kit (Qiagen). RNA concentration and integrity were assessed using the ND-1000 Spectrophotometer (NanoDrop). cDNA was synthesized using the Quantitect Reverse Transcription Kit (Qiagen). Reactions were carried out in triplicate with QuantiFast SYBR Green Master Mix (Qiagen) at an annealing temperature of 60 °C. Data were collected using the QuantStudio Real-Time PCR System Version 1.3 by Applied Biosystems. Primer identifying both long and short forms of prolactin receptor was designed using Primer Designing Software (NCBI) (https://www.ncbi.nlm.nih.gov/tools/primer-blast/); sequence: Forward: 5′ ATCTTTCCACCAGTTCCGGG, and Reverse: 5′ TTGGAGAGCCAGTCT CTAGC. Other primer sequences used in this study are as follows: for estrogen receptor 1 (ESR 1) : Forward: TCTGCCAAGGAGACTCGCTACT, and Reverse: 5′ GGTGCATTGGTTTGTAGC TGGAC; acetylcholinesterase (Ache)—Forward: 5′ TTCCTTCGTGCCTGTGGTAG, Reverse: 5′ CAGAAAGTAGGAGCCCTCGTC; BEN domain containing 5 (Bend5)—Forward: 5′ ACAACCAGAAGGTGTACGCG, Reverse: 5′ GTCAGA TTTGTCTTCTGCCAGC; cyclin G1 (Ccng1)—Forward: 5′ CACCTTGCCATTTGAGAG GAGA, Reverse: 5′ AGTGGCAGGCCTTAAGTTGG; 5-hydroxytryptamine receptor 3A (Htr3a)—Forward: 5′ CACACTCCTTCTGGGATACTCAG, Reverse: 5′ GCACAATGA AGATGGTCTCAGC; synaptotagmin 1 (Syt1)—Forward: 5′ TAGTCG TGACCTGCTGCTTC, Reverse: 5′ TCATCCTTAAGGGCCTGATCCT; phosphoglycerate kinase 1 (Pgk1)—Forward: 5′ TGCCAAGGCTTTGGAGAGTCC, Reverse: AAAGGCCATTCCACCACCAA. The relative amount of RNA was normalized to the expression of phosphoglycerate kinase 1 (Pgk1) mRNA as previously described^31^. Pgk1 was chosen as the reference gene because its expression was not affected by pregnancy or the treatment^32^. Gene expression level was calculated by the ∆∆Ct methods, relative to the expression level in islets of βPrlR^+/−^ mice on day 15 of pregnancy.

### RNAseq analysis

Total RNA (100–200 islets/mouse) was extracted using the RNeasy Mini Kit (Qiagen). The library preparation, quality control, sequencing and bioinformatic analysis generating differentially expressed transcripts was completed by UCDNA Services, Cumming School of Medicine, University of Calgary. RNA quality and quantity was measured on the Agilent 2200 TapeStation System. Only samples with a RNA integrity number above 7 were used. Messenger RNA was enriched and separated from rRNA using the NEBNextPoly(A) mRNA Magnetic Isolation Module (New England Biolabs). RNA library was prepared using the NEBNext Ultra II Directional RNA Library Prep Kit for Illumina (New England Biolabs). Sequencing was performed on Illumina NextSeq 550 System to obtain 51–62 million reads per sample. Reads were aligned with Kallisto 0.42.4^46^ to the mouse NCBI RefSeq transcript database (2017 version). Transcript counts were normalized as FPKM (fragments per kilobase of exon model per million reads mapped) and differential expression was detected with Sleuth (FDR < 0.05). The raw data sets discussed in this study have been deposited in the NCBI Gene Expression Omnibus and are accessible through GEO series accession number GSE156774 (https://www.ncbi.nlm.nih.gov/geo/query/acc.cgi?acc=GSE156774). Gene network analysis was performed using Qiagen’s Ingenuity Pathway Analysis platform (Qiagen N.V., Redwood City, CA) to identify pathways enriched. Enrichr, a web-based interactive enrichment analysis tool (https://maayanlab.cloud/Enrichr/) was also used to group these genes into functional categories, using the Kyoto Encyclopedia of Genes and Genomes (KEGG) pathway database and the Gene Ontology (GO) knowledgebase. The Heat map of differentially expressed transcripts was generated on the R console (version 3.4.3)^47^ by centering normalized transcript counts on the average count per transcript, and plotted with gplot (3.0.1)^48^ and RColorBrewer (1.1.2)^49^ packages.

### Statistical analysis

All statistics were performed using GraphPad Prism 6. Two-tailed Student’s t tests or ANOVA with Bonferroni post-tests were performed where appropriate. Comparisons were made between the heterozygous conditional knockout (βPrlR^+/−^), the wild type littermate (βPrlR^+/+^), or the Prlr^+/−^ mice, as stated in the Figure Legend.

## Supplementary Information

Supplementary Figure 1.

## Data Availability

The raw data set from the RNAseq experiment has been deposited in the NCBI Gene Expression Omnibus and are accessible through GEO series accession number GSE156774 (https://www.ncbi.nlm.nih.gov/geo/query/acc.cgi?acc=GSE156774).
