# Supplementary figures and images for: Beta cell adaptation to pregnancy requires prolactin action on both beta and non-beta cells
Source: Sci Rep. 2021 May 14;11:10372. doi: 10.1038/s41598-021-89745-9 (PMC8121891; doi:10.1038/s41598-021-89745-9)

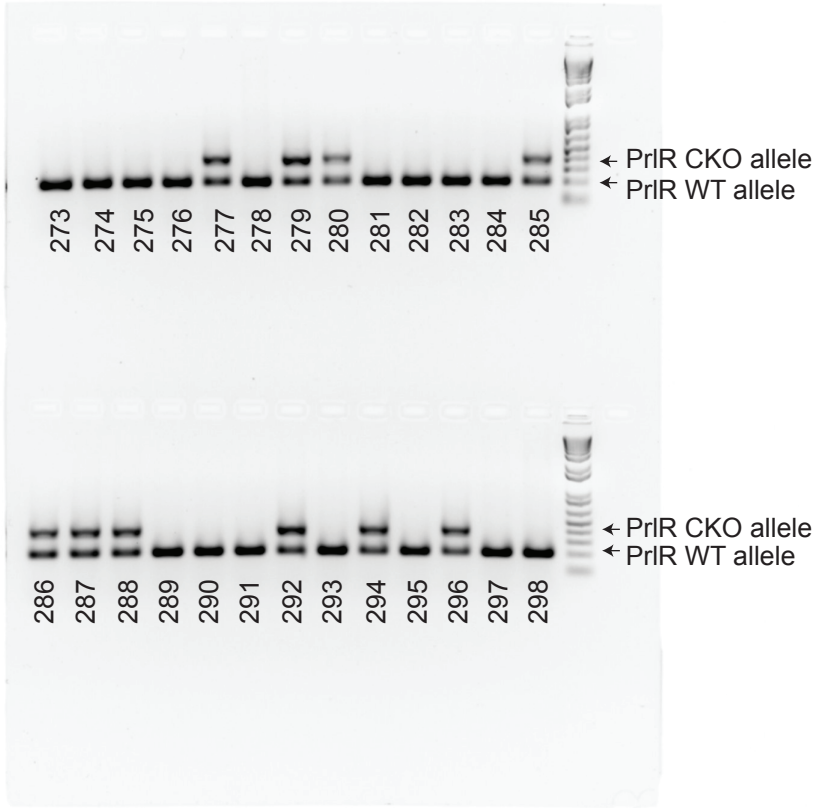

Supplement: Supplementary file 1 — Supplementary Figure 1. [file 41598_2021_89745_MOESM1_ESM.pdf]
